# Supplementary material for: Efficacy of Two Commercially Available Adsorbents to Reduce the Combined Toxic Effects of Dietary Aflatoxins, Fumonisins, and Zearalenone and Their Residues in the Tissues of Weaned Pigs
Source: Toxins (Basel). 2023 Oct 27;15(11):629. doi: 10.3390/toxins15110629 (PMC10675588; doi:10.3390/toxins15110629)
Supplement: Supplementary file 1 [file toxins-15-00629-s001.zip › toxins-2653312-supplymentary.pdf]

Supplementary Material

1. Take 1 g of a homogenized tissue (cut into pieces) sample and combine it with 200 µL of isotopic standard working solution;

2. Add 10 mL of acetonitrile/water/acetic acid (79:20:1, *v*/*v*/*v*);

3. Add 4.0 g anhydrous sodium sulfate and 1.5 g of anhydrous sodium acetate in a plastic tube (50 mL), and blend in a in ultra-turrax blender (IKA, T10) until completely dissolved;

4. Centrifuge at 5,000 rpm for 8 min;

5. Separate the supernatant and transfer it to another tube (50 mL);

6. Add 5 mL of hexane and vortex the mixture for 2 min;

7. Keep the mixture under dark for 5 min <u>BEFORE</u> hexane phase (upper phase) removal & discard;

8. Separate and transfer the acetonitrile phase into a glass tube following evaporation under flowing nitrogen gas (50 ± 5 °C) in a MultiVap 54 (LabTech) concentrator;

9. Redissolve the remaining dry residue in 0.5 mL of acetonitrile/water (10:90, *v*/*v*);

10. Vortex (30 s) the extract, filter through a 0.22 µm syringe filter and transfer the volume into an autosampler vial with an insert;

11. Submit a 5 µL aliquot to LC-MS/MS analysis.

Figure S1. Analytical steps for extraction of mycotoxin residues from samples of liver and kidneys.

Table S1. Analytical parameters of the analytical method for determination of residual mycotoxins in liver and kidneys.

Mycotoxin	RT (min.)	Mass (g/mol)	Molecular ion	Transition (*m/z*)	Calibration range (μg/kg)	LOD (μg/kg)	LOQ (μg/kg)
AFM_1	1.13	328.3	$[M+H]^+$	329.0 > 273.1 [a]<br>329.0 > 229.0 [b]	0.1-100	0.2	0.7
AFB_1	1.25	312.3	$[M+H]^+$	312.7 > 284.9 [a]<br>312.7 > 241.1 [b]	0.1-100	0.1	0.3
$[^{13}C_{17}]$-AFB_1	1.25	329.1	$[M+H]^+$	330.3 > 301.5			
AFB_2	1.15	314.3	$[M+H]^+$	314.7 > 259.0 [a]<br>314.7 > 287.0 [b]	0.1-100	0.1	0.4
AFG_1	1.18	328.3	$[M+H]^+$	328.9 > 243.0 [a]<br>328.9 > 199.5 [b]	0.1-100	0.2	0.6
AFG_2	1.16	330.3	$[M+H]^+$	330.9 > 245.0 [a]<br>330.9 > 188.9 [b]	0.1-100	0.2	0.8
$[^{13}C_{34}]$-FB_1	5.40	755.6	$[M+H]^+$	756.6 > 374.4			
FB_1	5.40	721.8	$[M+H]^+$	722.5 > 334.0 [a]<br>722.5 > 352.1 [b]	0.5-100	0.3	1.0
FB_2	5.76	705.8	$[M+H]^+$	706.5 > 336.2 [a]<br>706.5 > 318.3 [b]	0.5-100	0.4	1.0
$[^{13}C_{18}]$-ZEN	6.03	336.2	$[M-H]^-$	335.1 > 185.1			
ZEN	6.03	318.1	$[M-H]^-$	317.1 > 175.1 [a]<br>317.1 > 130.9 [b]	1.0-100	1.0	3.0
α-ZEL	5.81	320.2	$[M-H]^-$	319.1 > 275.2 [a]<br>319.1 > 160.2 [b]	1.0-100	0.8	2.6
β-ZEL	5.83	320.2	$[M-H]^-$	319.1 > 275.2 [a]<br>319.1 > 160.2 [b]	1.0-100	0.6	2.0

RT: Retention time; LOD: Limit of detection; LOQ: Limit of quantification; AF: aflatoxin; FB: fumonisin; ZEN: zearalenone; *α*-ZEL: *α*-zearalenol; β-ZEL: β-zearalenol.

[a] Transitions used in the quantification.

[b] Transitions used in the confirmation.
